# How Does Mangrove Restoration or Reforestation Change Trace Metal Pollution in Mangrove Ecosystems? A Review of Current Knowledge

**DOI:** 10.3390/toxics12110812

**Published:** 2024-11-13

**Authors:** Mohammad Mazbah Uddin, Kang Mei, Bin Xie, Li Cunlu, Shengxing Long, Fuliu Xu

**Affiliations:** 1 Key Laboratory of the Ministry of Education for Earth Surface Processes, College of Urban & Environmental Sciences, Peking University, Beijing 100089, China; 2206388553@pku.edu.cn (M.M.U.); 1700013217@pku.edu.cn (L.C.); longshengxing@163.com (S.L.); 2 Jiangsu Key Laboratory of Marine Bioresources and Environment, Jiangsu Institute of Marine Resources Development, Jiangsu Ocean University, Lianyungang 222005, China; meican@jou.edu.cn; 3 Key Laboratory of Marine Ecological Conservation and Restoration, Third Institute of Oceanography, Ministry of Natural Resources, Xiamen 361005, China; xiebin@tio.org.cn

**Keywords:** mangrove restoration, sediment properties, trace metals, organic carbon, current status

## Abstract

In recent years, mangrove restoration has gained significant attention due to its carbon storage capacity, role as a pollution sink, and ecological and economic benefits. Moreover, the United Nations Sustainable Development Goals’ strategies include mangrove restoration. This review investigates the status of mangrove restoration research and the influence of restoration on trace metal accumulation, speciation, and associated risks in mangrove sediments. Our analysis reveals that research on mangrove restoration is increasing globally, with a notable surge in publications after 2017. However, fewer than 25 articles published between 2007 and 2024 address trace metals in restored mangroves, indicating limited focus from researchers on this topic. Research shows that mangrove restoration can quickly alter sediment properties, such as texture, salinity, and pH. As a result, restored sediments tend to accumulate higher organic carbon content than barren areas. Most studies also suggest that trace metal accumulation is higher in restored sites than in non-restored areas. However, metal speciation varies considerably between sites due to local environmental factors. Furthermore, many studies suggest that restoration may increase the risks posed by trace metals to aquatic biota. The challenges of mangrove restoration research include short study timeframes, low success rates, poorly defined targets, small-scale efforts, conflicts with local communities over resources and benefits, insufficient government funding, and a lack of historical data on community health and pollution status.

## 1. Introduction

Mangrove ecosystems are diverse, highly productive, and carbon-rich intertidal forests, mostly found in the tropical, subtropical, and warm-temperate areas of the world [1]. These ecosystems provide valuable services, including coastal storm protection, habitat for fish, food sources for local communities, and carbon storage and sequestration, acting as sinks for and remediators of organic and inorganic pollutants [2,3,4,5]. Therefore, these ecosystems also provide cultural, spiritual, and leisure services, which significantly contribute to the economic benefits of local livelihoods [4]. This valuable ecosystem is highly threatened by anthropogenic stressors. A total of 62% of mangrove forests are lost globally due to land use changes such as conversion for aquaculture and agriculture and economic development in coastal areas [6]. Natural processes such as tsunamis and cyclones also contribute to global mangrove loss, but with a slower decline than anthropogenic loss [6]. The deforestation of mangroves increases coastal erosion, biodiversity fragmentation, and, most importantly, emissions of stock carbon dioxide under mangrove soils [7]. As we know, mangroves can store a higher amount of organic carbon in their sediments, and this emerging carbon is mostly deposited from woody biomass, dead wood biomass, root biomass, and leaf litter biomass through the process of soil carbon sequestration [8,9]. According to a recent study, global mangrove organic carbon stock is 1.93 pg, lower than previous estimations [10]. However, in the ecosystem (living and dead biomass, inorganic and organic carbon in sediment), carbon stock is estimated at 3.7–6.2 pg [10].

According to estimates, mangrove forests contribute 4.5 Mg C ha⁻¹ and 66.6 Tg C year⁻¹ to global mean net ecosystem productivity [11]. Consequently, the deforestation of mangroves leads to significant carbon emissions from mangrove soils, estimated at 23.5–38.7 Tg C year⁻¹ [10]. These rising carbon emissions contribute to global climate change. Therefore, the restoration and conservation of mangrove ecosystems are crucial strategies for securing the critical ecological benefits and economic services that they provide [12].

Globally, mangrove restoration and reforestation have garnered considerable attention due to their capacity for carbon storage, positioning them as a natural solution for climate change mitigation [11,13]. Recognizing the importance of ecosystem restoration, the United Nations declared 2021–2030 to be the UN Decade on Ecosystem Restoration [14]. Ecosystem restoration is a key component of the global Sustainable Development Goals [14]. Moreover, ecological restoration is essential for achieving climate change mitigation, poverty reduction, and biodiversity conservation on a global scale, making it a collective effort to restore degraded ecosystems and ensure a healthy planet for all [15].

In recent times, several countries of the world have committed to carbon neutrality (zero carbon emissions) to achieve the Paris Climate Agreement Goal of 1.5 °C warming by the end of this century [16]. For example, China will peak at its highest carbon emissions by the 2030s and be committed to carbon neutrality by the 2060s [16]. To achieve this Paris Climate Agreement Goal, a nature-based climate change solution is one strategy to conserve and restore blue-carbon ecosystems in coastal areas [16]. A recent study suggests that 118 TC can be restored in the blue carbon ecosystems on Chinese coastlines [16]. Moreover, in recent times, Indonesia has committed to restoring and planting mangroves of 6 million ha between the years 2020 and 2024 to achieve the Sustainable Development Goals [17].

Globally, the success rate of restoration is low, and the scale of restoration is still below the commitment [17]. But, globally, different organizations such as the IUCN, Mangrove Watch, and Mangrove Alliance are promoting mangrove conservation and restoration and providing funding for mangrove conservation research in different countries. In recent times, an IUCN mangrove research specialist group has been formed for conducting coordinated research on mangrove blue-carbon and restoration strategies [18]. As a result, different countries are proposing their plans for mangrove restoration and reviewing their existing plans for the better management of restored mangroves. But, large-scale mangrove restoration or reforestation may have other ecological consequences in coastal ecosystems. As we already know, mangroves can accumulate a higher amount of trace metals in their sediments than surrounding tidal flats or barren areas [19,20]. Several studies suggest that trace metals are a serious threat to mangrove ecosystems in Asia and several other mangrove-dominated countries in the world [21]. Therefore, mangrove ecosystems are refuges, especially for crabs, juvenile fishes, and benthic organisms, which are important for the functioning of this ecosystem.

Several studies on the influence of mangrove restoration or reforestation on trace metal accumulation, speciation, and mobility suggest that mangrove restoration can alter metal accumulation and speciation in mangrove sediments [19,20,22]. Restoration can also impact sediment properties, such as particle size, organic content, carbonate levels, and pH, which in turn affect metal speciation, accumulation, and mobility in mangrove ecosystems [19,20]. Some studies indicate that restored mangroves may exhibit higher trace metal accumulation in crabs and other benthic organisms compared to natural mangroves [19,20]. As mangrove restoration efforts increase globally, the potential impact on trace metal pollution becomes more significant. However, there is currently no comprehensive review examining how mangrove restoration affects metal pollution in coastal mangrove ecosystems, an essential gap for future restoration strategies and decision-making for conserving these valuable ecosystems.

The aims of this review are to assess the current state of research on mangrove restoration and trace metal pollution in restored or reforested mangrove ecosystems across different countries. This study also explores how mangrove restoration affects carbon storage and sequestration, sediment properties, metal accumulation, speciation, and mobility in these ecosystems. Additionally, it aims to identify potential research gaps and challenges in the restoration of mangrove ecosystems.

## 2. Research Status of Mangrove Restoration and Trace Metal Pollution

**Figure 1:**
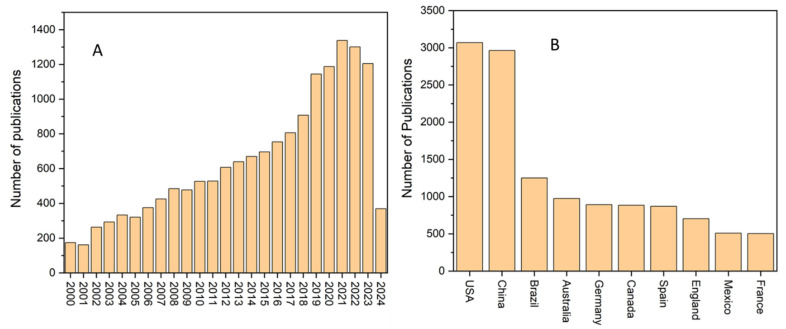
The number of research publications (**A**) and top ten countries (**B**) globally related to mangrove restoration between 2000 and 2024 (Data source: Web of Science).

For the analysis of global mangrove restoration/reforestation research status, we used the Web of Science core collection database to retrieve articles that were related to mangrove restoration research. We used the keyword “mangrove restoration or reforestation” in the search options of Web of Science. For increasing the credibility of articles, we only chose titles and keywords during the refining of articles with the mangrove restoration keyword [23]. This database showed that about 16,000 articles were published that were related to mangrove restoration between 2000 and 2024 [24] (Figure 1A).

After 2017, the number of research articles on mangrove restoration increased significantly compared to previous years, reflecting a growing interest in this field (Figure 1A). We also analyzed the countries contributing the highest number of publications. Figure 1B shows the top ten countries with the most research on mangrove restoration. The data suggest that the USA, China, and Brazil have produced more publications on mangrove restoration than other countries (Figure 1B).

**Figure 2:**
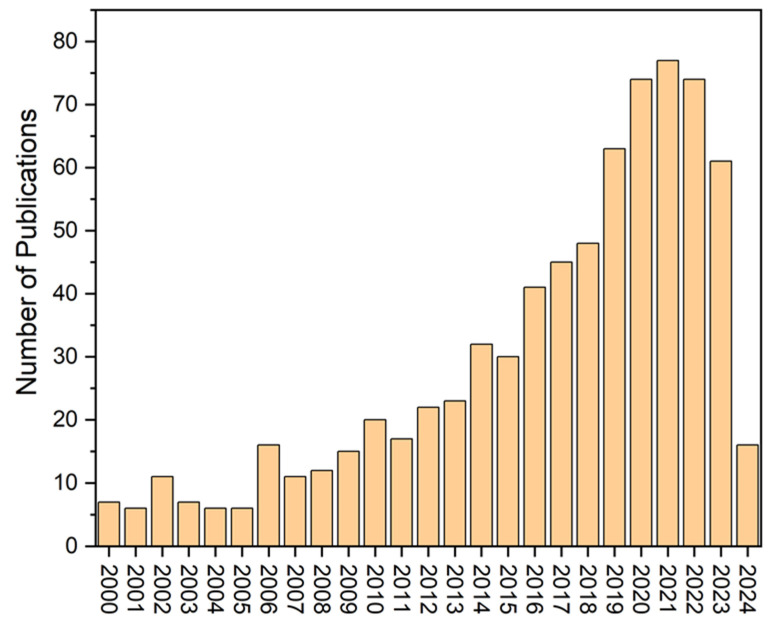
The number of research publications globally related to heavy metals in mangroves between 2000 and 2024 (Data source: Web of Science).

Therefore, we also used the keyword “heavy metals in mangroves” in the Web of Science database. We found that about 740 research articles were published between 2000 and 2024 (Figure 2).

Figure 2 also indicates that after 2018, research interest in trace metals within mangrove ecosystems increased significantly compared to previous years.

**Figure 3:**
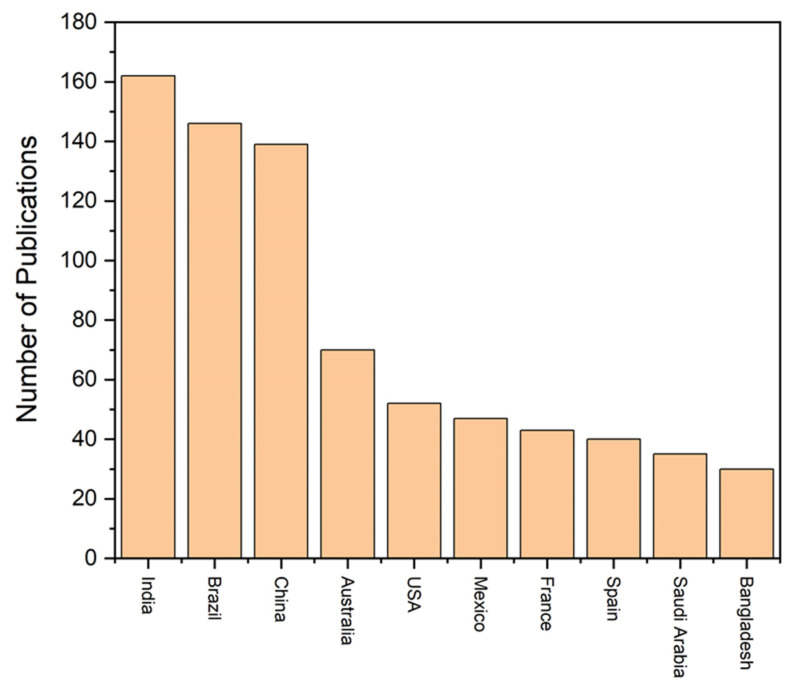
The top ten countries that published mangrove heavy metal publications in the world (Data source: Web of Science).

**Figure 4:**
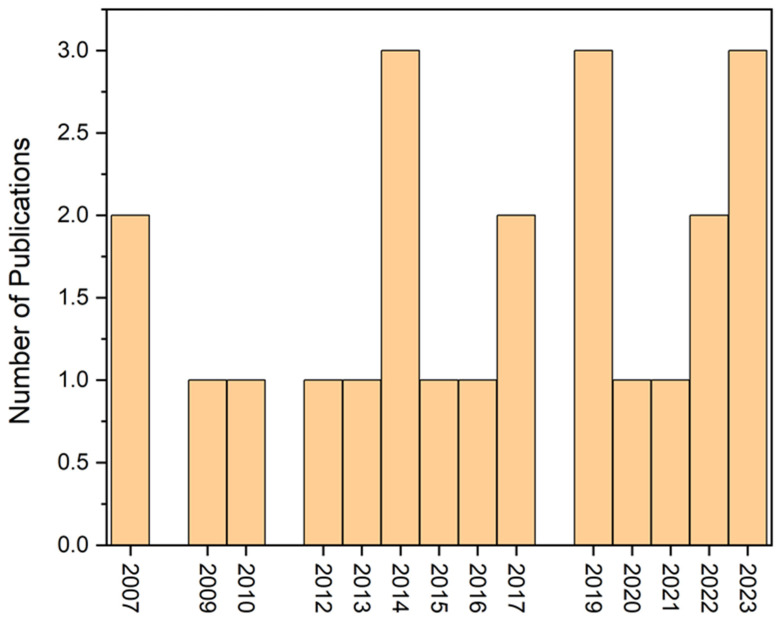
The number of research publications globally related to heavy metals in restored mangroves between 2007 and 2024 (Data source: Web of Science).

Therefore, Figure 3 indicates the top ten countries that contributed a higher number of mangrove trace metal research articles. Among the countries, India, Brazil, and China contributed a higher number of research articles than other countries (Figure 3). Moreover, we also used the keyword “heavy metals in restored mangroves” in the Web of Science database. We found that 23 research articles have been published between 2007 and 2024, which is significantly lower than the number for mangrove heavy metal research (Figure 2 and Figure 4).

However, these analyses suggest that heavy metals in restored mangroves research are afforded less concern by the scientific community. 

## 3. Reforestation/Restoration Influence on Sediment Physicochemical Properties

**Figure 5:**
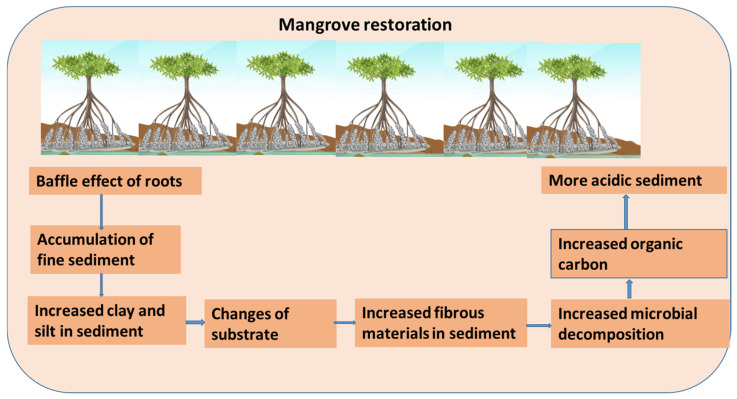
A schematic explanation of the influence of mangroves on sediment physicochemical properties.

In coastal and aquatic ecosystems, sediments are the main source and sink of pollutants [25,26,27,28]. Sediment physicochemical properties such as texture, salinity, and pH are crucial factors for heavy metal accumulation, speciation, binding, and transport to different environmental compartments of mangrove ecosystems [19,20,29,30,31]. Sediment texture (silt, minerals, and clay) has a close interaction with trace metal accumulation in the fluvial sediments [32,33,34,35,36]. However, sediment texture also indicates the particle size distribution and surface area of sediments, and those are essential parameters for the binding of trace metals to sediments [32,33,34,35,36]. But, several studies suggest that mangrove restoration can modify sediment properties such as texture, salinity, and pH very rapidly [19,20]. For instance, the restoration of the site of the Yifeng estuary contained higher finer (slit and clay) sediment than mud flats [19]. Moreover, mangrove planting could modify the sediment substrate through the accumulation of fine fibrous materials in sediments [37]. The changes in sediment physiochemical properties may also be related to the root density and planting density of mangrove species [37]. Therefore, the baffle effect of mangrove roots enhances the fine accumulation of sediments and stabilizes mangrove restoration areas [38] (Figure 5).

In an urban lagoon mangrove reforestation area, sediment texture was less fine than lagoon mud flat area [20]. This contrasting result from the lagoon mangrove reforestation sediment could have been influenced by the reduced natural tidal cycle and lack of river input to the mangrove areas [39,40]. A recent study suggests that the tidal cycle is related to the physiochemical properties of sediments and microbial diversity in mangrove sediments [39]. In a reforested mangrove area, the accumulation of higher fine-grained sediment was mostly of terrestrial origin; as a result, river input is very important for mangrove rehabilitation and establishment [39,40]. Several studies suggested that mangrove restoration can increase the acidification of sediment more than mudflats or surrounding barren areas [19,20]. This acidic condition is due to the increased microbial decomposition and oxidation of FeS and FeS_2_ [19,32].

## 4. Reforestation/Restoration Influence on Organic Content Storage and Sequestration

The coastal mangrove ecosystem is considered one of the most carbon-rich forests in the world [16]. It can sequester a high amount of carbon (both in below-ground and aboveground biomass) and is considered a nature-based solution for climate change mitigation and adaptation [16,41]. Several studies have suggested that mangrove restoration has increased organic matter content in the sediment of restoration sites than the surrounding barren areas [19,20,42]. The higher organic carbon content in restoration areas mostly originates from the decomposition of mangrove roots and litter [11]. Mangrove restoration can increase the anaerobic condition in the restoration sediment, which can facilitate the microbial decomposition of mangrove litter (Figure 5) [43]. Therefore, mangrove roots can diffuse oxygen and increase the oxidizing condition, which is important for the sequestration of organic carbon in sediment [19,43].

Reforestation has increased the microbial diversity and extracellular polymeric substances (EPSs) in microbial cells, which has influenced the microbial decomposition of mangrove roots, barks, and litters; as a result, higher organic carbon content has been produced and buried in mangrove sediment in the restoration areas [44]. Organic carbon storage capacity in restoration sites is related to the pattern of restoration in the coastal areas [45,46]. A recent study suggested that reforestation mangroves (where previously there were mangroves) had higher carbon storage levels than afforestation mangrove restoration sites. Higher organic carbon in reforestation sites is due to higher nitrogen content, lower salinity, and intertidal position [47]. The organic carbon sequestration in restoration mangrove areas is also related to species selection and restoration processes such as monoculture, mix culture, planting density, etc. [46]. Overall, it can be concluded that mangrove restoration increases organic carbon levels in restored sediments compared to mud flats. Moreover, mangrove species, plantation density, microbial diversity, and mangrove litter can facilitate the increase in organic carbon in restored sediments.

## 5. Reforestation/Restoration Influence on Metal Accumulation

**Figure 6:**
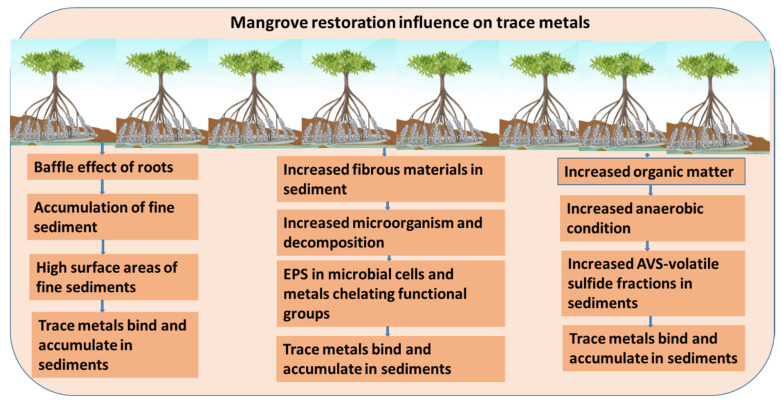
A graphical presentation of the influence of mangrove restoration on trace metal accumulation in mangrove sediments.

Globally, mangrove restoration or reforestation is increasing due to carbon storage, climate change mitigation, and ecosystem services. It is well established that mangrove restoration can facilitate the accumulation of higher amounts of trace metals in sediments than in surrounding non-mangrove areas’ sediments [19,20,47]. Several studies, such as those conducted in Yifeng Estuary, China; Yundang Lagoon, China; and Jinjiang Estuary, China, indicated that mangrove restoration sites had considerably higher trace metal accumulation than surrounding areas [19,20,48]. In the restoration areas of Yundang Lagoon sediments, the concentrations of Cu, Mn, and Pb were 59.82 µg/g, 996.54 µg/g, and 57.21 µg/g, but in mangrove barren areas, the concentrations were 54.41 µg/g, 584.34 µg/g, and 54.53 µg/g, respectively [20]. A graphical presentation of the influence of mangrove restoration on trace metal accumulation in sediments is displayed in Figure 6.

Several factors and mechanisms interplay with each other to influence metal accumulation, mostly in the upper layer of the mangrove restoration sediment [19,20,48]. Mangrove restoration increases the microbial decomposition of mangrove litter such as bark, roots, and leaves, which is also increasing the organic matter in sediments [49]. In addition, mangrove reforestation can enhance microorganisms, which can aggregate extracellular polymeric substances (EPSs) in their cell walls, and these substances have a significant influence on metal accumulations in sediments [44]. A study suggests that mangrove restoration increased EPSs production in the restoration areas, which have several metal-chelating functional groups to bind with trace metals in sediment and, ultimately, higher accumulation in reforestation sites [44,50]. Therefore, mangrove restoration significantly increases microorganisms, which also provide supporting roles for metal binding in sediment [50,51].

However, it is well known that mangrove forestation can increase fine particles and organic matter in sediments [26]. Fine particles and organic matter have higher specific surface areas where trace metals bind and accumulate in sediments [27]. Moreover, fine particles in mangrove areas can trap trace metals from overlaying water and transport them to deeper sediment layers [26,27]. Acid-volatile sulfide (AVS) fraction (reactive solid-phase sulfide fraction) is an important function of metal accumulation in sediment. In the mangrove restoration site, the AVS fraction in sediment is higher than in the mud flats [52]. These fractions can facilitate the binding of trace metals in sediment and act as an important carrier of trace metals in surface sediments [52]. The AVS fraction is also essential for increasing the anaerobic condition of restored mangrove areas [53]; as a result, organic matter is increased, which could cause metal accumulation in sediment.

## 6. Reforestation/Restoration Effect on Metal Speciation

In the ecological system, the speciation of trace metals mostly reflects metal species and their potential toxic effects on environment [53]. Therefore, speciation also indicates the geochemical process of trace metals and their transformation from one species to another in different environmental media [53]. Different environmental factors such as pH, redox potential, microbial mediation, organic and inorganic complexes, adsorption, filtration, and complexation are the inhabiting or accelerating factors for metal speciation processes in biological systems [54]. Plants mostly alleviate metal toxicity in the environment through the process of complexation and membrane barriers [54]. Several studies suggest that mangrove restoration area sediment have a lower acid-soluble fraction than the surrounding mud flats areas [19,20]. Among the metal fractions, the acid-soluble fraction has higher mobility and bioavailability than other fractions in sediments [27]. Reforestation of mangroves may facilitate the formation of metal-sulfide and metal–organic matter complexes, resulting in a lower acid-soluble fraction in mangrove sediment than in mangrove barren areas [19].

Mangroves can uptake and co-precipitate trace metals as Fe-Mn oxides, which contribute to lowering the bioavailable fractions of metals in reforested mangrove sediments [19]. In areas of artificial lagoon slugs, the reforestation of mangroves indicates that most studied metals are predominantly found in the residual fraction of both reforested mangrove and lagoon sediments [20]. Other fractions of trace metals also showed variability in concentrations in both reforested mangrove sediments and lagoon sediments [20]. Cd, Pb, As, and Zn were more highly bound in reducible fractions in reforested sediments than lagoon sediments [20]. Therefore, considering all studied metals, oxidizable fractions also had higher concentrations in mangrove restoration sediments than lagoon sediments, which also suggests that higher organic carbon in reforested sediments could be a possible scavenger of higher oxidizable fractions [20]. However, the soluble fraction in restored mangrove sediments was lower than in lagoon sediments, indicating reduced transport to adjacent ecosystems and lower bioavailability to aquatic organisms [20]. A recent study suggested that mangrove reforestation can enhance bacterial biodiversity and the production of extracellular polymeric substances (EPS) [44]. This study also found a negative correlation between bacterial diversity (EPS) and the bioavailability of trace metals (acid-soluble fraction) in reforested mangrove sediments [44].

Mangrove restoration increases organic carbon, which has a considerable influence on higher EPS production in bacterial cells. EPS has different functional groups that can bind with acid-soluble fractions and reduce the soluble form of trace metals in restored mangrove sediments [44]. Moreover, higher organic carbon and EPS in the mangrove restoration sediments may facilitate the formation of oxidizable fractions of metals, lowering the toxicity and bioavailability of trace metals in the reforested mangroves [44].

Another study in Jinjiang mangrove wetland suggests that the restoration of mangroves decreased the secondary phase fraction (acid-soluble, oxidizable, and reducible fractions) of Cu, Cr, Zn, and Pb by 41.31%, 22.89%, 27.06%, and 22.13%, respectively [37]. This result indicates that the restoration of mangroves can alleviate metal toxicity in the sediments of coastal areas. In the restoration sites, sediment pH, silt, clay, and sediment organic matter (SOM) are the main controlling factors of trace metal speciation in the Jinjinag restoration mangrove wetland [37]. In addition, the planting patterns of mangroves also have a considerable influence on metal speciation in restoration sites. This is owing to different plants having different releasing patterns of oxygen, rhizosphere soils, redox potential, and pH; those factors can influence the speciation patterns of trace metals in restored mangroves [37].

## 7. Reforestation/Restoration Influence on Heavy Metal-Related Risks to Aquatic Biota

Mangrove ecosystems are habitats for a higher number of aquatic organisms due to the greater amount of food sources and less predation from the higher-trophic-level organisms [55]. The dominant aquatic biota are fish and their juveniles, prawns, and crabs. Benthic organisms are also dominant in mangrove sediments due to tidal cycles and higher food sources [56]. The crabs are one of the most dominant and important species in mangrove ecosystems for their filtering capacity and burrowing in the mangrove sediments, which increases the presence of oxygen required for facilitating different chemical reactions [57,58]. Aquatic biotas are important for mangrove ecosystems’ food web structure. It is well known that mangrove aquatic biota can accumulate heavy metals in their tissues and biomagnify in their food web [30]. The risks of trace metals to aquatic biota are mostly related to the concentrations of metals in sediments [59]. The risks to aquatic organisms are also related to the bioavailable forms of metals in the sediments. The acid-soluble forms of metals have higher bioavailability than other metals in sediments and water, and only bioavailable forms are absorbed by aquatic organisms [34].

Several studies on mangrove ecosystems aquatic species suggest that aquatic species can accumulate higher amounts of trace metals, which pose considerable risks to aquatic biota [60,61]. But, in terms of chemical speciation, their fraction patterns represent changes in restored mangroves rather than natural mangroves [19,56]. The chemical speciation of trace metals is an important criterion for risks posed to aquatic biota in mangrove ecosystems. Moreover, the chemical speciation of heavy metals indicates the mobility and bioavailability of sediments, whose forms are ultimately available for aquatic organisms [27]. In the Yifeng Estuary areas, mangrove restoration sites had lower acid-soluble fractions than mud areas, signifying the reduced mobility and bioavailability of heavy metals in restored mangrove sediments [19]. Therefore, this less acid-soluble fraction in the restoration of mangrove sediments also indicates that heavy metals are less toxic to aquatic organisms than mudflat areas [19]. A similar result was also observed in Yundang Lagoon reforestation sites [20]. However, there are considerable variations in metal fraction concentrations between lagoons and restoration sites [20]. Overall, mangrove forestation in Yundang Lagoon reduced acid-soluble and reducible fractions of heavy metals, which suggests that mangrove sediments are less toxic to aquatic organisms than lagoon sediments [20].

But, considerable percentages of Cu, Pb, and Cd-bound acid-soluble fractions in restoration sediments are higher than in lagoon sediments, suggesting potential risks posed to aquatic biota [20]. Another study in the Jinjiang Estuary suggests that restoration increases the adverse toxicity of trace metals in surface and sub-surface sediments [52]. Therefore, Pb, Cu, and Ni had possible adverse effects on benthic organisms due to the higher threshold value for metal toxicity in restored mangroves than mudflat areas [52]. A recent study of urban restored mangroves in Australia indicated that crabs contained higher levels of trace metals in their tissues than in natural mangroves [62]. This research also suggests higher risks of crabs in restored mangroves than in natural mangrove sediments [62]. This higher risk of trace metals is also confirmed by the metabolomics profile of crab species, where higher amounts of proline and glutamate were found in crab tissues of restored mangroves than in natural mangroves [62]. The abundance of proline and glutamate in crab tissue indicates increases in trace metal stress and reactive oxygen species [62,63].

Overall, mangrove restoration can change the pollution status of trace metals, which may influence mangrove dominant species such as crabs and bivalves because these are indicator species of mangrove ecosystems. Therefore, restoration has changed the speciation of trace metals in sediments, as discussed earlier, but their risks to aquatic biota are also variable in different study sites [19,20,52]. This variation in trace metal speciation in different restoration sites suggests that local environmental factors, the age of restoration sites, the place of restoration such as urban areas, microbial diversity, river inputs, and the type of mangrove species may influence metal bioavailable forms in the sediments [19,20,47,50,52,62]. As a result, it can be concluded that restoration may increase the risks posed by trace metals to aquatic biota in the restoration sites than mudflats or mangrove barren areas.

## 8. Mangrove Restoration and Trace Metal Pollution Research Challenges and Recommendations

**Figure 7:**
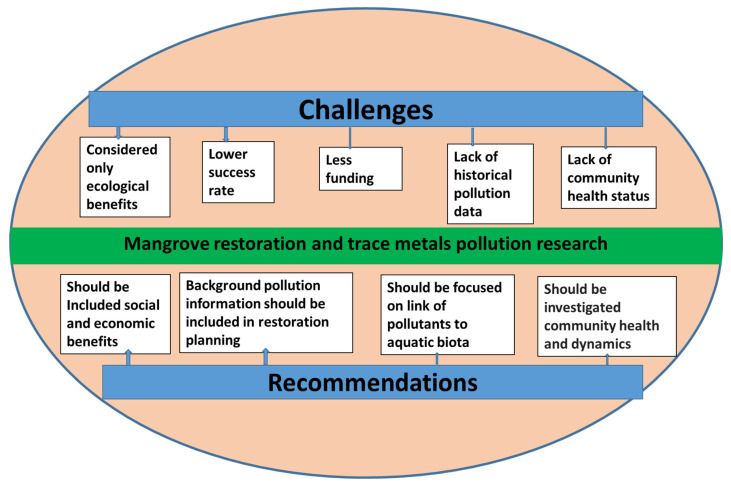
A graphical presentation of challenges and recommendations of mangrove restoration and trace metal pollution research.

Mangrove restoration is a complex decision-making process that requires adequate stakeholder participation for successful outcomes in terms of restoration benefits [64]. Previously, the success of restoration was mostly related to ecological benefits, where economic and social benefits were not taken into consideration [64]. But, for inclusive restoration, we must include economic and social benefits in restoration decision-making systems [65]. A graphical presentation of challenges and recommendations regarding mangrove restoration and trace metal pollution research is displayed in Figure 7.

In recent times, to achieve sustainable development goals, mangrove restoration has been an ambitious plan in most countries of the world. Mangrove restoration faces severe challenges in different countries due to their lower success rate, less determined target, small scales, conflict with residents for resources and benefits, and lower funding from government agencies (Figure 7) [17]. However, several studies indicated that mangroves can change the accumulation, speciation, behavior, and risks of trace metals in sediments of restoration areas [19,20,48,52]. However, the research publications on the influence of mangrove restoration on trace metal pollution are still very few. The challenges of this research are mostly related to the time frame, as the restoration needs a certain time to grow the forest. At most of the restoration sites, the initial background information such as sediment properties, organic carbon content, microbial diversity, metal accumulation, metal speciation, and possible ecological risks in sediments has not been quantified at the time of mangrove planting or restoration setting [20,48,52]. 

As a result of a lack of background information, researchers may face difficulties in drawing a conclusion about the influence of mangrove restoration on the pollution status of the restored sites. In these circumstances, we recommend that historical background information be included in the restoration decision and planning systems. In most trace metal research in restored or natural mangroves, surface sediment concentration is quantified. But, mangrove ecosystems contain a higher amount of aeration than other forests, resulting in higher variability in surface sediment trace metal concentrations [48]. In this context, restoration research may focus on core layers of sediments, which have more stable concentrations and can be evaluated or compared with historical background concentrations. Moreover, several studies indicated that mangrove restoration could increase the risks of trace metals to aquatic species in the restoration sites [19,20,62]. The challenges of risk evaluation at the restoration sites could be the possible lack of data about community fitness and community dynamics before the restoration activities [62]. The future research should focus on identifying the link between contaminants and aquatic organisms at the cellular level through metabolomics-based surveillance [62]. Therefore, ecotoxicological studies should include the comprehensive monitoring of post-restoration faunal health and outcomes. 

## 9. Conclusions

This review aims to explore the trends of mangrove restoration and trace metal pollution research in global mangrove ecosystems. A comprehensive analysis of the status of mangrove restoration and trace metal pollution research; the influence of restoration on sediment properties, trace metal accumulation, speciation, and risks in mangrove sediments; challenges; and recommendations are considered. According to the research status, mangrove restoration research has been increasing globally, and after 2017, this research increased significantly compared to previous years. But, in terms of restoration and trace metal pollution, fewer than 25 research articles have been published between 2007 and 2024, suggesting that less concern is being afforded to this issue by the scientific community. Mangrove restoration can change the sediment properties, including sediment texture, organic carbon, and pH, in restored areas compared to mangrove barren areas. Therefore, mangrove restoration could change the accumulation and speciation patterns of trace metals in the sediments of restoration sites due to changes in sediment properties and local environmental factors. In terms of ecological risks, mangrove restoration may increase the bioavailable forms of trace metals, which could increase the risks posed by trace metals to aquatic biota. Future research should include the historical pollution data of the restoration sites, status of pollution in different layers of sediments, community health and dynamics of ecosystems, and metabolomics status of aquatic biota before restoration.

## Data Availability

All the information and data are included in this paper.
